# Beyond the Asylum Walls: Tracing the Tapestry of Mental Health Interventions Across Eras and Cultures

**DOI:** 10.7759/cureus.48251

**Published:** 2023-11-04

**Authors:** Prachi Gupta, Komal N Muneshwar, Anup Juganavar, Tejas Shegekar

**Affiliations:** 1 Medicine, Jawaharlal Nehru Medical College, Datta Meghe Institute of Higher Education and Research, Wardha, IND; 2 Community Medicine, Jawaharlal Nehru Medical College, Datta Meghe Institute of Higher Education and Research, Wardha, IND

**Keywords:** psychiatry, cultural considerations, contemporary approaches, historical perspectives, interventions, mental health

## Abstract

This article offers an extensive review of the changing field of mental health therapies, charting a transformational path from traditional methods to modern breakthroughs and speculating on potential future developments. The story develops by investigating historical viewpoints while reflecting on the present and highlighting the lessons learned and their impact on contemporary practices. We have advanced from the stigmatized constraints of asylums to a paradigm that puts human rights, dignity, and individualized, culturally sensitive treatment first. Modern methods are much more varied and evidence-based, from cutting-edge technical advancements to evidence-based psychotherapies. The ethical considerations arising from the delicate balance of pharmacological therapies underline the responsibility of administering drugs that significantly affect mental health. Cultural factors become a pillar, highlighting how crucial cultural sensitivity is to promoting tolerance. By acknowledging how many facets of the human experience are interrelated, holistic methods help close the gap between the mind and body. Integrative medicine and alternative therapies represent a shift away from reductionist approaches and toward a holistic viewpoint. The delivery of mental health treatment is being reimagined by technological advancements, with virtual and digital environments opening up new access and support channels. These developments cut beyond regional boundaries, reinventing conventional therapy dynamics and paving the way for individualized therapies. Cultural concerns highlight the significance of cultural competency in navigating the complex mental health treatment system and adapting interventions to fit the particular requirements of various cultural contexts. With telepsychiatry, virtual reality, and artificial intelligence among the new technologies that promise to further revolutionize mental health therapies, the essay looks to the future. This review concludes by imagining a day when mental health is prioritized, therapies are available, and the diversity of human experience is valued. The path to a society that values, nurtures, and celebrates mental health continues.

## Introduction and background

The search for effective therapies has never been more crucial, given the dynamic state of global mental health. It is essential to critically evaluate current techniques, and foresee future paths in mental health therapies as we negotiate the complex web of psychological well-being. The prevalence of mental health issues is shown by the World Health Organization estimates that one in four individuals may have mental or neurological diseases at some time in their lives [1]. Although fundamental, traditional psychotherapy paradigms are under investigation, and innovation thrives on many fronts. The delivery of mental health treatment is changing due to the incorporation of new therapy modalities, technology, and a greater emphasis on cultural competency. Recent research emphasizes the importance of digital mental health therapies and shows how they may improve accessibility and offer affordable solutions [2]. Artificial intelligence (AI) development in mental health is significant, with sophisticated algorithms demonstrating promise in accurate diagnosis and individualized treatment planning [3].

Additionally, the return of psychedelic-assisted treatments is forcing a paradigm shift in the profession and offering fresh perspectives for studying and treating mental health illnesses [4]. It is crucial to consider the cultural subtleties affecting mental health treatment as we begin this research on present trends and future directions in interventions for mental health. There is a clear need for culturally competent strategies considering different origins and viewpoints [5]. This assessment combines data from various sources, including novel research and current advancements. This article presents a thorough review of mental health therapies by critically analyzing the available research, providing insights into the difficulties encountered and the bright future that lies ahead.

## Review

**Figure 1:**
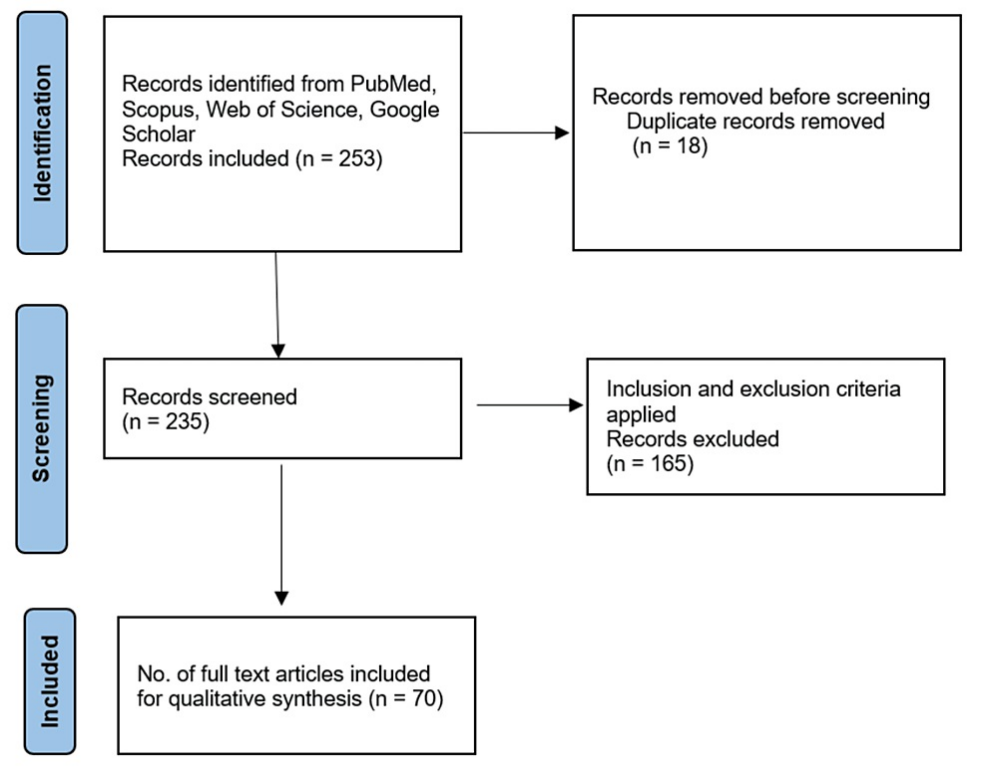
The selection process of articles used in this study

For this narrative review, a comprehensive search strategy was implemented across key databases, namely PubMed, Scopus, Web of Science, and Google Scholar. The search encompassed literature published from the year 1975 until the present, ensuring a thorough exploration of historical, contemporary, and emerging perspectives on mental health interventions. The search terms utilized a combination of controlled vocabulary (MeSH terms) and free-text terms related to mental health, interventions, historical perspectives, contemporary approaches, cultural considerations, and emerging trends. Inclusion criteria encompassed articles focusing on mental health interventions, historical perspectives, contemporary approaches, cultural considerations, and future directions, while exclusion criteria were applied to eliminate studies unrelated to the scope of mental health interventions. The screening process involved a two-stage approach, with initial screening based on titles and abstracts, followed by a thorough assessment of full-text articles against inclusion/exclusion criteria. A total of 70 articles meeting the inclusion criteria underwent a rigorous review process. To enhance transparency and replicability, the flow diagram has been included, detailing the selection process and the number of records at each stage (Figure 1). This methodology ensures the reliability and credibility of the synthesized information presented in this narrative review.

Historical perspectives

Tracing the Evolution of Mental Health Interventions: From Asylums to Modern Therapies

Significant changes have occurred throughout the historical course of therapies for mental health, reflecting evolving societal perceptions, and growing scientific knowledge. In the premodern age, asylums were frequently used to house people with mental health issues. Bethlem Royal Hospital in London is one such example [6]. Instead of therapeutic involvement, the emphasis was on confinement. Movements for moral treatment began to take shape in the late 18th century, led by reformers like Philippe Pinel and William Tuke. These groups promoted more compassionate and humane methods, highlighting the significance of environmental variables in mental health [7]. However, psychodynamic approaches, influenced by Sigmund Freud's psychoanalysis, were not widely used until the middle of the 20th century. The focus switched to investigating how early events and the unconscious mind affect mental health [8]. The development of psychopharmacology in the second part of the 20th century sparked a revolution. Introducing drugs like imipramine and chlorpromazine changed the therapeutic environment by providing pharmacological therapies for various mental diseases [9].

A paradigm shift occurred toward more varied and individualized treatments as the twenty-first century emerged. Cognitive-behavioral therapy (CBT) and other evidence-based psychotherapies have become more widespread [10]. Technology has also made creative interventions like virtual therapy and mobile mental health applications possible. In summary, the development of mental health therapies shows a shift from institutional care to a multifaceted and individualized strategy. Every historical stage, from the time of the asylum to the modern era of various treatment methods, has helped shape the current mental health interventions field.

Contemporary approaches

The Diverse Tapestry of Mental Health Interventions: A Comprehensive Overview

Diversity is the distinguishing feature of today's mental health therapies, reflecting the realization that no approach suits everyone. A thorough review reveals a diverse tapestry of treatment approaches, from conventional psychotherapy to cutting-edge technology advancements. The cornerstone of treatment for improving mental health outcomes, CBT, emphasizes the interaction of ideas, feelings, and behaviors [10]. A comprehensive approach to mental health is ensured by the persistence of psychodynamic treatments founded on understanding unconscious processes alongside more recent versions like mentalization-based treatment [11].

Technology integration has sparked a paradigm change in the provision of mental health services. Individuals seeking help have new and convenient options thanks to digital mental health interventions, such as mobile applications, websites, and virtual reality (VR) therapy [12]. AI and machine learning are also progressing, allowing for personalized treatments by analyzing large databases to customize therapeutic approaches based on distinct patient needs and responses [13].

Additionally, the resurgence of psychedelic-assisted treatments has drawn attention and threatened traditional therapy approaches. It has been shown that drugs like psilocybin and 3,4-methylenedioxymethamphetamine (MDMA) can treat diseases, including depression, anxiety, and post-traumatic stress disorder (PTSD) [14]. These methods show that the mind-body link and the study of altered states of consciousness are once again prioritized in treating mental illness. Contemporary approaches in medical interventions are summarized in Table 1 [3,10,15].

**Table 1:** Contemporary approaches to mental health interventions Reference: [3,10,15] Credit: The table was created by the authors. AI: Artificial intelligence

Year	Intervention approach	Key trends and innovations
2010	Telepsychiatry	Remote mental health services, increasing access
2015	Cognitive behavioral therapy	Evidence-based therapy, emphasis on cognition
2020	Mindfulness interventions	Integration of mindfulness practices in treatment
2023	AI	AI-driven tools for personalized mental health care

Trends Shaping the Present: A Deep Dive into Current Approaches

Numerous significant themes define contemporary mental health practices and influence the current environment. The focus on cultural competency recognizes that people come from different origins and experiences, and it promotes solutions dedicated to these differences [5]. Therapies that are culturally appropriate guarantee that mental health services are accessible to all populations and efficient. With a move toward detecting and managing mental health disorders at its beginning, preventive methods and early treatments are becoming increasingly critical. To lessen the long-term burden of mental illness, school-based programs, business initiatives, and community outreach programs all attempt to build environments that promote mental well-being from the beginning [16]. The importance of peer support and community engagement in mental health treatment is increasingly being acknowledged. Peer-led therapies and support groups give people with lived experiences a forum to interact, communicate, and exchange knowledge, developing a feeling of community and comprehension [17].

Pharmacological interventions

Psychiatric Medications: Balancing Benefits and Challenges in Mental Health Treatment

How mental health is treated has changed dramatically due to advancements in pharmacotherapy. These drugs have helped numerous people suffering from mental health illnesses, from the early discoveries of antipsychotics and antidepressants to the creation of more focused treatments. For ailments including depression and anxiety, doctors frequently prescribe antidepressants like selective serotonin reuptake inhibitors (SSRIs) and serotonin-norepinephrine reuptake inhibitors (SNRIs). They function by altering the brain's neurotransmitter levels, successfully reducing symptoms, and fostering emotional well-being [18]. Antidepressant usage, however, is not without obstacles; prominent problems in clinical practice include tolerability, side effects, and the delay in the beginning of therapeutic benefits [19].

Lithium and anticonvulsant drugs are important mood stabilizers in the treatment of bipolar illness. Although careful monitoring is necessary owing to potential adverse effects such as renal impairment, lithium has been a staple in avoiding manic and depressive episodes [20]. Today, various illnesses, including bipolar disorder and several depressive disorders, are treated with antipsychotic drugs, formerly primarily recommended for schizophrenia. Although effective, second-generation antipsychotics have concerns regarding metabolic adverse effects, such as weight gain and an increased risk of diabetes [21]. Although they offer quick relief from acute anxiety symptoms, benzodiazepines and other anxiolytics carry the danger of dependency and withdrawal. An ongoing problem in treating anxiety disorders is balancing the short-term advantages with the long-term hazards [22].

An atypical antipsychotic drug called clozapine is a last-resort therapy for schizophrenia. Its effectiveness is unrivaled, but the risk of agranulocytosis demands strict monitoring, illustrating the tight balance between therapeutic advantages and significant side effects [23]. While there is no doubt that these drugs have revolutionized mental health care, concerns remain regarding the over-reliance on pharmaceutical therapies, possible side effects, and the requirement for more individualized treatment methods. Healthcare professionals must consider the bigger picture of a patient's mental health, combining psychotherapy, lifestyle changes, and psychosocial support to maximize general well-being.

Innovations in Pharmacotherapy: Exploring the Future of Psychiatric Medications

Exciting breakthroughs poised to overcome present drawbacks and improve treatment results will shape the future of psychiatric drugs. Pharmacogenomics, a young discipline, promises the potential of customizing psychiatric medications based on a person's genetic profile. This individualized strategy maximizes therapy response, reduces side effects, and enhances general safety [24]. Depression therapy has advanced thanks to N-methyl-D-aspartate (NMDA) receptor modulators like ketamine. For those who have failed to respond to traditional treatments, ketamine has been shown to provide solid and quick antidepressant effects [25]. New mechanisms of action in psychiatric pharmacotherapy are being explored by investigating additional glutamatergic drugs. A growing body of research is focusing on inflammation as a cause of mental health issues. For their potential to lessen the symptoms of depression and other mood disorders, antiinflammatory medications such as cytokine modulators and nonsteroidal antiinflammatory drugs are being researched [26]. This represents a shift away from earlier neurotransmitter-centered strategies.

Furthermore, those who do not react to medicine alone have options; thanks to developments in the field of neurostimulation, such as transcranial magnetic stimulation (TMS) and electroconvulsive treatment (ECT). These therapies alter brain networks and neurotransmitter systems, giving patients with illnesses resistant to therapy more alternatives [27]. As we speak about the future of psychiatric drugs, the need to lower stigma, improve accessibility, and consider a person's overall well-being remains crucial. The key to improving mental health outcomes in the following years will be to combine pharmaceutical therapies with a thorough treatment strategy that tackles socioeconomic determinants of health and integrates a variety of therapy modalities.

Technological innovations

Digital Mental Health: Revolutionizing Access and Support

The development of digital mental health treatments as transformational instruments has revolutionized the accessibility and provision of mental health support. Harnessing technology's capacity for mental health in a time when it permeates every aspect of our lives is both novel and essential. One of the most accessible digital mental health help types is smartphone applications. Applications include various services like virtual therapy sessions, mindfulness exercises, and mood tracking. For instance, research has examined the efficacy of mobile applications in easing the symptoms of anxiety and depression [28]. These treatments, which are frequently accessible around the clock, enable people to practice self-care whenever it suits them.

Many people use online counseling services to engage with qualified mental health specialists virtually. Online therapy is effective, according to studies that show equivalent results to in-person sessions [29]. This eliminates geographical boundaries while addressing some areas' lack of mental health experts. Digital mental health now heavily relies on telepsychiatry, which includes videoconferencing for psychiatric appointments. Telepsychiatry makes specialized care more accessible and is especially important in rural or disadvantaged regions. Studies have shown that it is helpful in various contexts, including treating PTSD and enhancing medication compliance [30]. Digital mental health solutions use AI to provide individualized and data-driven therapies. Machine learning algorithms analyze large-scale datasets to forecast trends in mental health, pinpoint risk factors, and customize treatments for different people. Chatbots and virtual assistants powered by AI also offer real-time assistance and information. AI bots can breach confidentiality by mishandling or inadequately securing sensitive user data, raising concerns about privacy in digital mental health interventions. The necessity for evidence-based practices, data protection, and ethical concerns are still paramount as we explore the digital frontier of mental health. Even if there are significant potential advantages, continuing study and watchfulness are crucial to ensuring digital technology's proper and moral application in mental health treatment [3].

Beyond Reality: The Therapeutic Potential of Virtual Environments in Mental Health

Formerly exclusive to the entertainment industry, virtual worlds are increasingly used as robust mental health therapeutic tools. These immersive technologies go beyond conventional treatment approaches and provide fresh approaches to various mental health issues. VR therapy is becoming more well-known as a cutting-edge treatment for various mental health conditions. Exposure treatment in VR is beneficial in treating phobias, PTSD, and social anxiety [31]. In a therapeutic context, people may confront and negotiate their concerns by re-enacting experiences in a controlled and adaptable atmosphere. VR is progressing in the treatment of psychosis as well. Virtual simulations give people who are exhibiting psychotic symptoms the chance to interact in a secure and monitored setting, offering opportunities for cognitive reorganization and improving coping skills [32]. This novel strategy has the potential to support more conventional psychosis therapies.

Applications for mindfulness and relaxation in virtual worlds help people manage their emotions and reduce stress. People can explore tranquil settings and engage in mindfulness exercises through guided VR experiences and biofeedback systems, which promote relaxation and resilience [33]. It is also being investigated how augmented reality (AR) may be used therapeutically. AR applications provide dynamic and individualized experiences by superimposing digital information onto the physical environment. AR has been applied to mental health to improve psychoeducation by giving people with illnesses like schizophrenia visual and interactive content to help them understand complex ideas [34]. Although virtual environments have the potential to be therapeutic, more study is needed to determine their effectiveness, safety, and ethical implications. To ensure that virtual technologies are responsibly included in mental health treatment, accessibility, affordability, and user experience must be carefully considered.

Cultural Considerations

Cultural Competence in Mental Health Interventions: Navigating Diversity and Inclusion

More people realize the need for cultural competency in offering inclusive and flourishing mental health interventions. Building trust, enhancing treatment results, and addressing inequities in mental health, it is essential to comprehend and navigate the many cultural backgrounds of those seeking mental health help. Research emphasizes the need for cultural competency training to improve mental health practitioners' awareness, knowledge, and abilities in working with varied populations [35]. The impact of cultural influences on people's perceptions of mental health, behavior associated with seeking assistance, and treatment choices is something that culturally competent practitioners are aware of.

Recognizing the cultural stigma associated with mental health is crucial to cultural competency. Different cultures and societies have different stigmatizing views toward mental illness. Culturally sensitive mental health interventions are more likely to connect with people and address the particular problems that stigma presents [5]. An expanding field of study focuses on how evidence-based therapies might be culturally tailored. Studies examining the efficacy of culturally tailored treatments for various mental health issues have demonstrated that these interventions are more successful in terms of engagement and results when specifically tailored to a particular cultural setting [36]. This strategy ensures that mental health treatments are acceptable to varied populations and appropriate to their needs.

Effective communication and comprehension in mental health settings depend heavily on language. To meet the linguistic demands of varied populations, culturally competent interventions consider language diversity and offer resources and services in several languages [37]. This strategy improves accessibility and makes sure that individuals receive mental health treatment without being hindered by language problems. Fostering a therapeutic partnership based on respect and trust is another critical aspect of providing mental health care that is culturally competent. A collaborative approach that respects the viewpoints of people from various cultural backgrounds helps to foster a healthy therapy relationship and encourages improved treatment adherence [38].

Finally, it should be noted that cultural competency is a continuous commitment to comprehending and valuing the diversity of people who need mental health treatment. We can create a mental health system that is more inclusive and fairer by promoting cultural competency in mental health services. Cultural considerations in mental health are summarized in Table 2 [35,39-41].

**Table 2:** Cultural considerations in mental health interventions Reference: [35,39-41] Credit: The table was created by the authors.

Year	Cultural competence approach	Key considerations
2000	Culturally tailored therapies	Addressing specific cultural beliefs and practices
2010	Language accessibility	Providing services in clients' preferred languages
2020	Community engagement	Involving local communities in mental health initiatives
2022	Intersectionality	Recognizing the impact of intersecting identities

To ensure that therapies are successful, considerate, and inclusive, it is crucial to customize mental health methods to match the particular demands of various cultural settings. Beyond recognizing diversity, cultural tailoring entails customizing treatments to fit cultural values, beliefs, and practices. Cultural humility, a crucial element of individualized mental health therapies, emphasizes a lifelong dedication to self-reflection, learning, and respectful collaborations with people from other origins [42]. Practitioners who take a culturally humble perspective can better comprehend the subtleties of various cultural settings and jointly build solutions that connect with people's specific experiences.

Individuals' opinions on mental health are frequently and significantly influenced by their religious and spiritual beliefs. For engagement and efficacy, interventions must be specifically designed to reflect and respect these ideas. As an illustration, faith-based therapies have been investigated as responsive, culturally appropriate methods for addressing mental health issues within religious groups [43]. In many cultural situations, the family and the community are essential elements. Customized mental health therapies may include using family-based strategies, neighborhood support systems, and integrating elders or community leaders in the therapy process. These tactics acknowledge the relationship between more comprehensive social and cultural systems and mental health [44].

The selection of therapy methods can also be tailored culturally. Cultural relevance and acceptability can be increased by incorporating traditional healing modalities into mental health therapies, such as storytelling, art therapy, or indigenous healing rites [45]. This method acknowledges and honors many perspectives on understanding and advancing mental health. It is crucial for cultural tailoring to take socioeconomic issues into account. Economic inequalities, immigration-related stresses, or other social factors that disproportionately impact a particular cultural group may require tailored treatments to be addressed [46].

In conclusion, adapting mental health methods to different cultural contexts requires a comprehensive awareness of cultural values, beliefs, and practices. Culturally appropriate therapies support diversity, honor the particular experiences of each person, and support more fair and effective mental health care.

Preventive strategies

Proactive Measures for Mental Wellness: Early Interventions and Preventive Strategies

Implementing early treatments and preventative measures to advance mental well-being and lower the risk of mental health problems is part of the transition in mental health care toward a proactive strategy. Research has shown that risk factors must be addressed, and protective ones must be promoted early on to promote robust mental health throughout the lifespan. Early interventions in schools promote children's and teenagers' mental health. Preventing mental health problems is helped by school-based initiatives that emphasize resilience development, coping skills instruction, and mental health education [16]. These treatments are designed to give young people the skills to successfully deal with pressures and obstacles.

Programs for parent education have shown promise in halting the emergence of behavioral and emotional issues in kids. Programs that teach parents how to be good parents, communicate effectively, and control their emotions help to create a nurturing home environment and lower the likelihood of mental health issues in kids [47]. Preventive measures also apply to work environments, where stress and burnout can worsen mental health conditions. Proactive steps to avoid mental health issues at work include stress management classes, employee help programs, and supportive work environments [48]. Employers may support their workers' overall mental well-being by managing pressures and fostering a pleasant workplace culture. The more significant public health approach to mental well-being includes community-based preventative initiatives focusing on particular groups or addressing common problems. For instance, the incidence of mental health issues in a community can be dramatically impacted by interventions that concentrate on preventing drug misuse, preventing suicide, and building community resilience [49]. The creation of digital tools for mental health illustrates how technology is being used in preventative methods. Early treatments and preventive mental health care are accessible and scalable through mobile applications, internet platforms, and telehealth services [12]. These sites include ways to manage stress, track moods, and engage in guided treatments that advance mental well-being.

In conclusion, preventive measures for mental well-being entail a multifaceted strategy considering various environments and life phases. The reactive model of mental health treatment may give way to a preventative paradigm by investing in early interventions, educating people, and building supportive settings.

Breaking the Cycle: Examining the Role of Prevention in Mental Health Care

With the chance to act early and halt the development of diseases, prevention is essential to ending the cycle of mental health issues. Examining risk factors, fostering resilience, and putting solutions into practice at many societal levels are just a few facets of prevention's varied role in mental health treatment.

Before the appearance of symptoms, risk factors are addressed as part of primary prevention, which aims to lower the prevalence of mental health disorders. Primary prevention is aided by public awareness efforts that target mental health, de-stigmatize it, and encourage positive lifestyle choices [50]. By fostering a supportive atmosphere for mental health, these initiatives will lessen the overall burden of mental health problems. Early diagnosis and intervention for those at risk of developing mental health illnesses are considered secondary preventative measures. Screening programs, especially in primary care settings, aid in spotting people who are showing the first indications of mental health problems [50]. Early intervention can enhance long-term results and stop symptoms from getting worse.

By recognizing and treating mental health problems in children and adolescents, school-based mental health programs help with secondary prevention. This at-risk group can have fewer mental health issues if psychoeducation, counseling, and supportive educational environments are implemented [51]. Tertiary prevention concentrates on lessening the effects of mental health illnesses that have already developed and preventing relapses. Tertiary prevention relies heavily on having access to quick and efficient treatment, continuous support, and rehabilitation programs [52]. This entails supporting long-term healing and ending the cycle of repeated episodes.

Holistic approaches

Integrative Medicine and Mental Health: Bridging the Gap Between Mind and Body

A paradigm change in mental health treatment is represented by integrative medicine, which emphasizes the connection between the mind and body to advance general health. This strategy goes beyond conventional approaches by including complementary and alternative therapies with orthodox psychiatric interventions. A complex interplay between biological, psychological, and social elements is addressed by integrative medicine, according to research, which can improve the outcomes for mental health. Integral parts of integrative treatment for mental health include mind-body techniques like yoga and mindfulness meditation. By encouraging self-awareness, emotional control, and a sense of inner peace, these practices have effectively lowered symptoms of anxiety, depression, and stress [53]. Incorporating mind-body therapies into psychiatric treatment recognizes the reciprocal link between physical and mental health.

Another area of integrative medicine that acknowledges how diet affects mental health is nutritional psychiatry. A growing body of research indicates that eating habits high in nutrients, such as omega-3 fatty acids, vitamins, and minerals, might affect neurotransmitters and help prevent and treat mental health illnesses [54]. Including dietary therapies in treatment strategies for mental illness emphasizes the comprehensive character of mental health. An old Chinese practice known as acupuncture has gained popularity as a supplemental method of providing mental health care. According to studies, acupuncture may modify neurotransmitter systems, lessen inflammation, and treat depression and anxiety symptoms [55]. Acupuncture is a prime example of the integrative philosophy that considers physical and mental dimensions by treating energy imbalances inside the body. A holistic approach to patient treatment is provided through collaborative care models, which pair integrative medicine experts with traditional mental health practitioners. With the help of a multidisciplinary team, individuals are guaranteed to get thorough, individualized therapies that cover the whole range of their mental health requirements [56]. To promote a more inclusive and patient-centered approach to mental health treatment, integrative medicine helps to close the gap between the mind and the body.

Holistic Healing: A Comprehensive Approach to Mental Well-Being

Considering how many facets of the human experience are interrelated, holistic healing in mental health goes beyond the traditional medical approach. This all-encompassing strategy acknowledges that elements affecting mental health go beyond symptoms and diagnoses, including lifestyle choices, social support, and the integration of the mind, body, and spirit.

Holistic healing includes therapeutic methods such as dance movement therapy, music therapy, and art therapy. These artistic methods offer opportunities for emotional processing, self-expression, and a more profound awareness of one's inner reality [57]. Holistic medicine encourages people's creative and expressive sides while embracing a variety of nonverbal modes of communication. Integrative healing relies heavily on mindfulness-based therapies rooted in traditional contemplative practices. The practice of mindfulness fosters present-moment acceptance and the ability to observe thoughts and emotions without passing judgment. According to research, mindfulness-based techniques can lessen depressive and anxious symptoms, improve general psychological health, and boost quality of life [58]. Mindfulness in treating mental illness is consistent with the holistic approach to treating the mind-body relationship.

The social aspect of holistic therapy emphasizes the value of a sense of belonging and interpersonal connections for mental health. Peer support programs, community involvement, and support groups all work to build a feeling of community and lessen social isolation. This collective strategy acknowledges that people are part of more significant social circumstances that greatly influence their mental health [59]. In holistic therapy, environmental variables are also considered, recognizing the impact of physical environments on mental health. A holistic healing process benefits from therapeutic settings encouraging relaxation, connection to nature, and sensory stimulation [60]. The inclusion of environmental factors is consistent with the notion that the environment influences mental states.

Challenges and critiques

Ethical Dilemmas in Mental Health Interventions: Navigating Complex Terrain

It is difficult for practitioners to negotiate the ethical atmosphere of mental health therapies because of its many problems. Issues such as confidentiality violations, informed consent, and cultural competency must be carefully considered to preserve ethical standards. Additional topics, including data privacy, security, and the proper application of AI, are brought up by the junction of technology and mental health [61]. The ethical ramifications of coercive interventions, including required hospital stays or therapy, also spark discussions about autonomy and human rights [62]. It is consistently challenging to balance beneficence and independence, especially when personal preferences could contradict the perceived effectiveness of a specific course of therapy. Since ethical issues are ever-evolving, mental health workers must get moral training. This ongoing training should consider new technology and societal trends [63].

Beyond Praise: Addressing Criticisms and Identifying Opportunities for Improvement

Although there have been tremendous advancements in mental health therapies, obstacles and critiques still exist, calling for a critical evaluation of current procedures. The reevaluation of treatment modalities is prompted by worries about the overuse of psychotropic medicines, which may have long-term adverse effects and overdiagnosis problems [64]. Accessibility still poses a severe problem since marginalized populations frequently lack access to high-quality mental health care [65]. Critiques also highlight the interventions' inadequate cultural sensitivity, highlighting the demand for more inclusive strategies. Given the prevalence of individual-focused therapy, it is unclear if cultural and structural influences on mental health are being neglected. In the future, there are areas where improvements might be made to integrate various therapy modalities, addressing societal determinants of mental health, and creating a collaborative, interdisciplinary approach to care. Adopting patient-centered and culturally competent practices guarantees that therapies are efficient, fair, and sensitive to the various needs of people [35].

Future directions

Emerging Trends: Shaping the Future Landscape of Mental Health Interventions

Emerging trends that use cutting-edge technologies and treatment strategies are influencing the direction of mental health interventions in the future. For instance, with the growth of telepsychiatry, people may now obtain mental health care from a distance. In addition to addressing accessibility concerns, this movement creates new opportunities for ongoing monitoring and individualized treatments via digital platforms [66]. Additionally, coming soon are VR and AR, which will provide immersive experiences for exposure treatment, cognitive training, and cutting-edge therapeutic modalities [33].

The mental health treatment field is entirely transformed by AI. Machine learning systems analyze enormous datasets to find trends, forecast treatment outcomes, and offer tailored suggestions. The development of AI-driven chatbots and virtual assistants to provide real-time assistance and interventions might improve accessibility and lessen the stigma associated with asking for help [3]. Another recent development that shows promise for personalized therapies is incorporating genetics into mental health care. A personalized approach to treatment based on unique genetic profiles is made possible by understanding the genetic underpinnings of mental health disorders. Particularly in the context of pharmacogenomics, the goal is to optimize the choice and administration of medications to enhance therapeutic efficacy and lessen side effects [67].

Personalized Medicine in Mental Health: A Glimpse into Tomorrow's Practices

With the advent of personalized medicine, the future of therapies for mental health is on the verge of a paradigm change. The potential for improving therapeutic results in mental health care is enormous when treatments are adapted to patient traits, preferences, and genetic composition.

Pharmacogenomics, which uses genetic data to forecast a person's reaction to psychiatric drugs, is one approach to personalized medicine. Based on a person's genetic profile, this method seeks to discover the most effective treatments with the fewest adverse effects [68]. Personalized medicine improves treatment efficacy and lessens the burden for people with mental health issues by reducing the trial-and-error process in drug administration. The application of biomarkers as a decision-making tool for therapy is a further aspect of personalized medicine in mental health. Specific biomarkers linked to many mental health diseases may now be identified because of molecular biology and neuroimaging developments. These biomarkers may help with diagnosis, prognosis, and therapy selection, opening the door for focused therapies [69].

They have personalized mental health treatment benefits from using digital health technology. Real-time data on people's behaviors, physiological reactions, and everyday activities may be collected using wearable technology and smartphone applications. Clinicians can develop individualized and effective therapies using this data analysis to gain a thorough picture of a patient's mental health condition [70]. Ethics, data privacy, and interdisciplinary cooperation will be essential in navigating this revolutionary context as we advance toward personalized medicine in mental health. Future mental health therapies will be precisely tailored to each person's particular requirements, thanks to the convergence of genetic knowledge, biomarkers, and digital technology. A summary of studies included in this review is mentioned in Table 3.

**Table 3:** Summary table of studies included in the review

Sr no	Study title	Authors	Year	Type of study	Summary
1	Mental health [1]	World Health Organization	2023	Review	Overview of mental health by the World Health Organization
2	Machine learning, statistical learning and the future of biological research in psychiatry [3]	Iniesta R, Stahl D, McGuffin P	2016	Review	Exploring the future of biological research in psychiatry using machine learning
3	Psilocybin with psychological support for treatment-resistant depression: an open-label feasibility study [4]	Carhart-Harris RL, Bolstridge M, Rucker J, et al.	2016	Feasibility study	Investigating the use of psilocybin for treatment-resistant depression
4	Toward a new architecture for global mental health [5]	Kirmayer LJ, Pedersen D	2014	Perspective	Proposing a new architecture for global mental health
5	From madness to mental illness: medical men as moral entrepreneurs [6]	Scull AT	1975	Historical analysis	Examining the transition from madness to mental illness
6	A history of psychiatry: from the era of the asylum to the age of Prozac [7]	Ford, Charles V. Editor; Margolese, Ellen BA, LLB, MD	2000	Historical review	Tracing the history of psychiatry from the era of the asylum to the age of Prozac
7	On the history of psychiatry [8]	Edwards ML, Magoon C	2021	Historical overview	Providing insights into the history of psychiatry
8	Current status of benzodiazepines [9]	Greenblatt DJ, Shader RI, Abernethy DR	1983	Review	Reviewing the current status of benzodiazepines
9	The efficacy of cognitive behavioral therapy: a review of meta-analyses [10]	Hofmann SG, Asnaani A, Vonk IJ, Sawyer AT, Fang A	2012	Meta-analysis	Evaluating the efficacy of cognitive-behavioral therapy through a review of meta-analyses
10	Digital mental health and COVID-19: using technology today to accelerate the curve on access and quality tomorrow [12]	Torous J, Jän Myrick K, Rauseo-Ricupero N, Firth J	2020	Commentary	Exploring the role of digital mental health during COVID-19
11	Cure therapeutics and strategic prevention: raising the bar for mental health research [13]	Insel TR, Scolnick EM	2006	Perspective	Discussing cure therapeutics and strategic prevention in mental health research
12	Mindfulness based interventions in context: past, present, and future [15]	Kabat-Zinn J	2003	Perspective	Reflecting on the past, present, and future of mindfulness-based interventions
13	The Gatehouse Project: can a multilevel school intervention affect emotional wellbeing and health risk behaviours? [16]	Bond L, Patton G, Glover S, Carlin JB, Butler H, Thomas L, Bowes G	2004	Intervention study	Investigating the impact of a multilevel school intervention on emotional well-being and health risk behaviors
14	World Federation of Societies of Biological Psychiatry (WFSBP) guidelines for biological treatment of unipolar depressive disorders, part 1: update 2013 on the acute and continuation treatment of unipolar depressive disorders [19]	Bauer M, Pfennig A, Severus E, Whybrow PC, Angst J, Möller HJ	2013	Treatment guidelines	Updating guidelines for the biological treatment of unipolar depressive disorders
15	Treatment of bipolar disorder [20]	Geddes JR, Miklowitz DJ	2013	Review	Reviewing the treatment of bipolar disorder
16	Prevalence, incidence and mortality from cardiovascular disease in patients with pooled and specific severe mental illness: a large-scale meta-analysis of 3,211,768 patients and 113,383,368 controls [21]	Correll CU, Solmi M, Veronese N, et al.	2017	Meta-analysis	Investigating the prevalence, incidence, and mortality of cardiovascular disease in severe mental illness
17	Evidence-based guidelines for the pharmacological treatment of anxiety disorders: recommendations from the British Association for Psychopharmacology [22]	Baldwin DS, Anderson IM, Nutt DJ, et al.	2005	Treatment guidelines	Providing evidence-based guidelines for the pharmacological treatment of anxiety disorders
18	Clinical Pharmacogenetics Implementation Consortium guideline for CYP2D6 and CYP2C19 genotypes and dosing of tricyclic antidepressants [24]	Hicks JK, Swen JJ, Thorn CF, et al.	2013	Guideline	Providing guidelines for CYP2D6 and CYP2C19 genotypes and dosing of tricyclic antidepressants
19	The effect of a single dose of intravenous ketamine on suicidal ideation: a systematic review and individual participant data meta-analysis [25]	Wilkinson ST, Ballard ED, Bloch MH, et al.	2018	Meta-analysis	Investigating the effect of intravenous ketamine on suicidal ideation through a systematic review and meta-analysis
20	Effect of anti-inflammatory treatment on depression, depressive symptoms, and adverse effects: a systematic review and meta-analysis of randomized clinical trials [26]	Köhler O, Benros ME, Nordentoft M, Farkouh ME, Iyengar RL, Mors O, Krogh J	2014	Meta-analysis	Analyzing the effect of antiinflammatory treatment on depression through a systematic review and meta-analysis
21	Can smartphone mental health interventions reduce symptoms of anxiety? A meta-analysis of randomized controlled trials [28]	Firth J, Torous J, Nicholas J, Carney R, Rosenbaum S, Sarris J	2017	Meta-analysis	Evaluating the effectiveness of smartphone mental health interventions in reducing symptoms of anxiety
22	Virtual reality exposure therapy in anxiety disorders: a quantitative meta-analysis [31]	Opriș D, Pintea S, García-Palacios A, Botella C, Szamosközi Ş, David D	2012	Meta-analysis	Quantitatively analyzing the utility of virtual reality exposure therapy in anxiety disorders
23	Virtual reality in the assessment, understanding, and treatment of mental health disorders [33]	Freeman D, Reeve S, Robinson A, Ehlers A, Clark D, Spanlang B, Slater M	2017	Review	Discussing the role of virtual reality in the assessment, understanding, and treatment of mental health disorders
24	Future directions: how virtual reality can further improve the assessment and treatment of eating disorders and obesity [34]	Gutiérrez-Maldonado J, Wiederhold BK, Riva G	2016	Perspective	Exploring the potential of virtual reality in improving the assessment and treatment of eating disorders and obesity
25	Rethinking the concept of acculturation [37]	Schwartz SJ, Unger JB, Zamboanga BL, Szapocznik J	2010	Perspective	Rethinking the concept of acculturation in the context of mental health
26	Factors contributing to optimal human functioning in people of color in the United States [38]	Constantine MG, Sue DW	2006	Review	Examining factors contributing to optimal human functioning in people of color in the United States
27	Development and factor structure of the cross-cultural counseling inventory [39]	LaFromboise TD, Coleman HLK, Hernandez A	1991	Instrument development	Developing and exploring the factor structure of the Cross-Cultural Counseling Inventory
28	Narrative and discursive approaches to the analysis of subjectivity in psychotherapy [40]	Avdi E, Georgaca E	2009	Theoretical analysis	Applying narrative and discursive approaches to analyze subjectivity in psychotherapy
29	Interest in spiritually integrated psychotherapy among acute psychiatric patients [43]	Rosmarin DH, Forester BP, Shassian DM, Webb CA, Björgvinsson T	2015	Survey	Investigating interest in spiritually integrated psychotherapy among acute psychiatric patients
30	Gender, family, and community correlates of mental health in South Asian Americans [44]	Masood N, Okazaki S, Takeuchi DT	2009	Correlational study	Examining gender, family, and community correlates of mental health in South Asian Americans
31	American Indian and Alaska Native mental health: diverse perspectives on enduring disparities [45]	Gone JP, Trimble JE	2012	Perspective	Presenting diverse perspectives on enduring disparities in American Indian and Alaska Native mental health
32	Rethinking cultural competence [46]	Kirmayer LJ	2012	Perspective	Rethinking the concept of cultural competence in mental health
33	Can work make you mentally ill? A systematic meta-review of work-related risk factors for common mental health problems [48]	Harvey SB, Modini M, Joyce S, et al.	2017	Meta-review	Conducting a systematic meta-review of work-related risk factors for common mental health problems
34	Suicide research, prevention, and COVID-19 [49]	Niederkrotenthaler T, Gunnell D, Arensman E, et al.	2020	Review	Discussing suicide research, prevention, and the impact of COVID-19
35	Clinical diagnosis of depression in primary care: a meta-analysis [50]	Mitchell AJ, Vaze A, Rao S	2009	Meta-analysis	Conducting a meta-analysis on the clinical diagnosis of depression in primary care
36	Unmet need for mental health care among U.S. children: variation by ethnicity and insurance status [51]	Kataoka SH, Zhang L, Wells KB	2002	Epidemiological study	Investigating unmet needs for mental health care among US children, with variation by ethnicity and insurance status
37	Yoga, mindfulness-based stress reduction and stress-related physiological measures: a meta-analysis [53]	Pascoe MC, Thompson DR, Ski CF	2017	Meta-analysis	Conducting a meta-analysis on the effects of yoga and mindfulness-based stress reduction on stress-related physiological measures
38	Nutritional psychiatry: the present state of the evidence [54]	Marx W, Moseley G, Berk M, Jacka F	2017	Review	Presenting the present state of evidence in nutritional psychiatry
39	Lifestyle medicine for depression [56]	Sarris J, O’Neil A, Coulson CE, Schweitzer I, Berk M	2014	Review	Discussing lifestyle medicine for the treatment of depression
40	Peer support/peer provided services underlying processes, benefits, and critical ingredients [59]	Solomon P	2004	Review	Exploring the underlying processes, benefits, and critical ingredients of peer support/peer-provided services
41	Mental health courts and the complex issue of mentally ill offenders [62]	Watson A, Hanrahan P, Luchins D, Lurigio A	2001	Perspective	Discussing mental health courts and the complex issue of mentally ill offenders
42	The role of culture and cultural techniques in psychotherapy. A critique and reformulation [63]	Sue S, Zane N	1987	Theoretical analysis	Critiquing and reformulating the role of culture and cultural techniques in psychotherapy
43	Telepsychiatry for mental health service delivery to children and adolescents [66]	Kommu JV, Sharma E, Ramtekkar U	2020	Review	Discussing telepsychiatry for mental health service delivery to children and adolescents
44	Pharmacogenomic testing and personalized treatment of depression [68]	Perlis RH	2014	Review	Discussing pharmacogenomic testing and personalized treatment of depression

## Conclusions

The condition of mental health treatments today results from a remarkable journey characterized by innovation, transformation, and a rising dedication to holistic well-being. Since mental health treatment has historically only been provided in asylums, society has advanced toward a time where mental illness is no longer stigmatized, diversity is celebrated, and mental health is considered essential to total wellness. The emphasis on human rights, dignity, and the need for individualized, culturally competent care results from modern practices that rely on historical teachings. In the current environment, mental health interventions weave a rich tapestry that recognizes the complexity of mental health and the uniqueness of each experience. This ranges from cutting-edge technical advancements to evidence-based psychotherapies and pharmaceutical treatments. Pharmaceutical treatments, a mainstay of contemporary mental health care, cause people to consider how to weigh the advantages and disadvantages. The development of new psychiatric drugs shows promise, but ethical concerns and continuous monitoring are essential to ethical prescribing procedures.

Cultural factors become crucial, highlighting the significance of cultural competency in mental health therapies. Acknowledging diversity and adapting strategies to particular cultural situations is vital for providing inclusive, efficient care. The increasing significance of preventive techniques signals a move toward proactive interventions for mental well-being. Examining the importance of early intervention and prevention in ending the cycle of mental health problems is part of this strategy. By acknowledging the interconnection of multiple facets of human experience, holistic methods close the mind-body gap. Integrative medicine is a break from reductionist approaches and embraces a holistic viewpoint. It includes mind-body techniques and alternative therapies. The delivery of mental health treatment is being reimagined by technological advancements, with virtual and digital environments opening up new access and support channels. By rethinking conventional treatment dynamics, these innovations cut through regional boundaries. The significance of cultural competency is further emphasized by cultural concerns when navigating the vast field of mental health care. Strategies must be tailored to the particular requirements of various cultural situations to promote inclusion. Future prospects for treatments in mental health hold forth even more promising opportunities. AI, VR, and other new developments, such as telepsychiatry, have the potential to revolutionize mental health treatment further. In conclusion, the changing landscape of therapies for mental health is a journey from seclusion to integration and from stigma to acceptance. As we look to the future, we see a world where mental health is taken seriously, solutions are available, and the diversity of human experience is valued. The path toward a society that treats, nurtures, and celebrates mental health continues.
